# Application of Data Science in Circulating Tumor DNA Detection: A Promising Avenue Towards Liquid Biopsy

**DOI:** 10.3389/fonc.2021.692322

**Published:** 2021-07-21

**Authors:** Ming Li, Sisi Xie, Chenyu Lu, Lingyun Zhu, Lvyun Zhu

**Affiliations:** Department of Biology and Chemistry, College of Liberal Arts and Sciences, National University of Defense Technology, Changsha, China

**Keywords:** cancer diagnosis, ctDNA detection, data science, liquid biopsy, technological advancement

## Abstract

The circulating tumor DNA (ctDNA), as a promising biomarker of liquid biopsy, has potential clinical relevance on the molecular diagnosis and monitoring of cancer. However, the trace concentration level of ctDNA in the peripheral blood restricts its extensive clinical application. Recently, high-throughput-based methodologies have been leveraged to improve the sensitivity and specificity of ctDNA detection, showing a promising avenue towards liquid biopsy. This review briefly summarizes the high-throughput data features concerned by current ctDNA detection strategies and the technical obstacles, potential solutions, and clinical relevance of current ctDNA profiling technologies. We also highlight future directions improving the limit of detection of ctDNA for better clinical application. This review may serve as a reference for the crosslinks between data science and ctDNA-based liquid biopsy, benefiting clinical translation in advanced cancer diagnosis.

## Introduction

Liquid biopsy, a non-invasive real-time method, can provide diagnostic and prognostic information during cancer progression and treatment (1). Unlike tissue biopsy, liquid biopsy examines circulating tumor cells (2) or tumor-released molecules, such as DNAs (3) and RNAs (4), from the circulatory system. Circulating tumor DNA (ctDNA) is generated from tumor cells (5), which forms a small minority of the cell-free DNA (cfDNA) in circulation against a background of fragments mostly derived from normal cells in the event of cell death or exosome secretion (6, 7). Plasma ctDNA could originate from both the nuclei or the mitochondria of tumor cells (8). However, only nucleus ctDNA records sufficient information of tumor genome, revealing tumor generation, development, metastasis, and recurrence (9), while mitochondrial ctDNA often provides information noise due to its less genomic information and higher copy number (**Supplementary Figure 1**). Thus, the concentration and abnormal sequence features of nucleus ctDNA (hereinafter ctDNA for convenience) in patients’ blood are significantly correlated with the course of the disease and curative effect (10), rendering it an emerging tumor marker and an essential part of liquid biopsy (11). Although the trace concentration of ctDNA in the peripheral blood and intense background noises challenge the clinical application of ctDNA, a series of ctDNA capture methods based on data science aiming at its biological features improves the sensitivity and accuracy of ctDNA detection and gradually clears the obstacles in the potential clinical application of ctDNA detection (12, 13). This review briefly summarizes the recent development and application of data science for highly sensitive and robust ctDNA detection. We also discuss the current challenges of ctDNA detection technologies and provide insights into the potential development direction in their future application.

## Data Features Utilized by Current ctDNA Detection Strategies

Current ctDNA detection strategies are developed mainly based on the fragment concentration and the sequence features, such as abnormal mutations and methylations. The dynamic concentration of ctDNA is significantly correlated with the progress of the cancer disease. Because of its short half-life of less than 2 hours (14) and its low concentration (5), ctDNA is almost undetectable in patients with primary tumors. However, along with the progression of the disease, the immune system is attenuated, and ctDNA is gradually accumulated, which could be discriminated from cfDNA under certain limits of detection (15). The increased concentration of ctDNA could identify patients with cancer from healthy cohorts and stratify patients in the early and advanced stages (16). Besides, the changes in ctDNA levels before and after drug treatment are related to the therapeutic effect for patients (17). Furthermore, for tumor-free patients after treatment, the concentration level of ctDNA indicates the risk of cancer recurrence (18).

Technically, concentration analysis of ctDNA is a challenging task because ctDNA makes up a small proportion of the total cfDNA extracted from serum (19, 20). For example, Diehl et al. found that the mean mutated allele frequency of *APC* gene of patients with colorectal cancer ranged from early stages’ 0.04% to late stages 11% (21). Extraction of ctDNA information from other cfDNA noise should be an initial step for ctDNA detection. Size selection–based data selection has been widely utilized in ctDNA detection to increase the signal-to-noise ratio. The size of cfDNA generated from the apoptosis of normal cells is about 167 bp, which is due to the structure of the histone octamer (22). However, studies show that the ctDNA is shorter than cfDNA in the meaning of statistics (23) and has a typical size of less than 142 bp in low molecular weight (24). The enriched mitochondrial ctDNA is about 100 bp in size, much smaller than that of nuclear ctDNA, further displaying the size variety of ctDNA fragments (8). Notably, there are long-size cfDNA fragments that exist, such as 2 kb and 20 kb fragments, which are probably generated from cancer cell necrosis (25) and blood cell surface (26), respectively. The accurate enrichment of ctDNA in a particular interval size eliminates some background noises to some extent and relatively enhances data processing efficiency. For example, Mouliere et al. mapped the distribution of ctDNA fragments and optimized the ctDNA capture by choosing to concentrate ctDNA fragments in the size of 90–150 bp from the blood samples (27). In parallel, the interference from mitochondrial ctDNA fragments can be significantly eliminated by reducing the interval size of captured DNA fragments (28, 29), and the size selection strategy not only greatly reduces the cost of sequencing but also considerably decreases the false-positive rates of results by data analysis (30).

The sequence information carried by ctDNA can reflect the mutation load and the methylation features of tumor cells. ctDNA profiling facilitates their delineation not only on a genome-wide scale but also in some specific genes or intervals of the tumor genome. Some studies showed that the mutational spectrum constructed by ctDNA is highly consistent with that of tissue biopsy (31, 32). Besides, ctDNA, which comes from a broader range of tumor cells, represents the heterogeneity of the tumor mutational spectrum better than the tissue biopsy (31). The spatial heterogeneity with the tumor’s continuous self-cloning and the temporal heterogeneity possibly resulting from drug resistance can be tracked by real-time monitoring in ctDNA fragments (33). An inspiring technology termed Cancer Personalized Profiling by deep sequencing (CAPP-Seq) preselected some specifically mutational exon regions by mining a large number of genetic mutations *in silico*. These exon-containing ctDNAs are subsequently extracted from serum cfDNAs using customized probes and then analyzed by high-throughput sequencing. This method could remarkably improve the detection sensitivity and specificity of ctDNAs by reducing the potential impact of stochastic noise and biological variability (34) (**Supplementary Figure 2**).

In addition, the methylation features of ctDNA reveal some epigenetic information of cancer patients. The methylation patterns can be maintained stably throughout the life span after *de novo* methylation (35), and the changes in the methylated patterns predict the risk of diseases (36). Because of the increased accuracy of high-throughput sequencing (HTS) technologies, the slight differences in methylation profiles between cancer patients and healthy cohorts shed light on the differential gene expression patterns at the epigenetic level and the relevance of epigenetic modification and tumor stage (37). For instance, the hypermethylation of tumor suppressor genes is strongly consistent with cancer occurrence, indicating that the methylation status of these modifications detected by ctDNA can play an essential role in the early detection of cancer and the determination of tissue of origin, and those patterns benefit machine learning for classification modeling (38).

**Figure 1:**
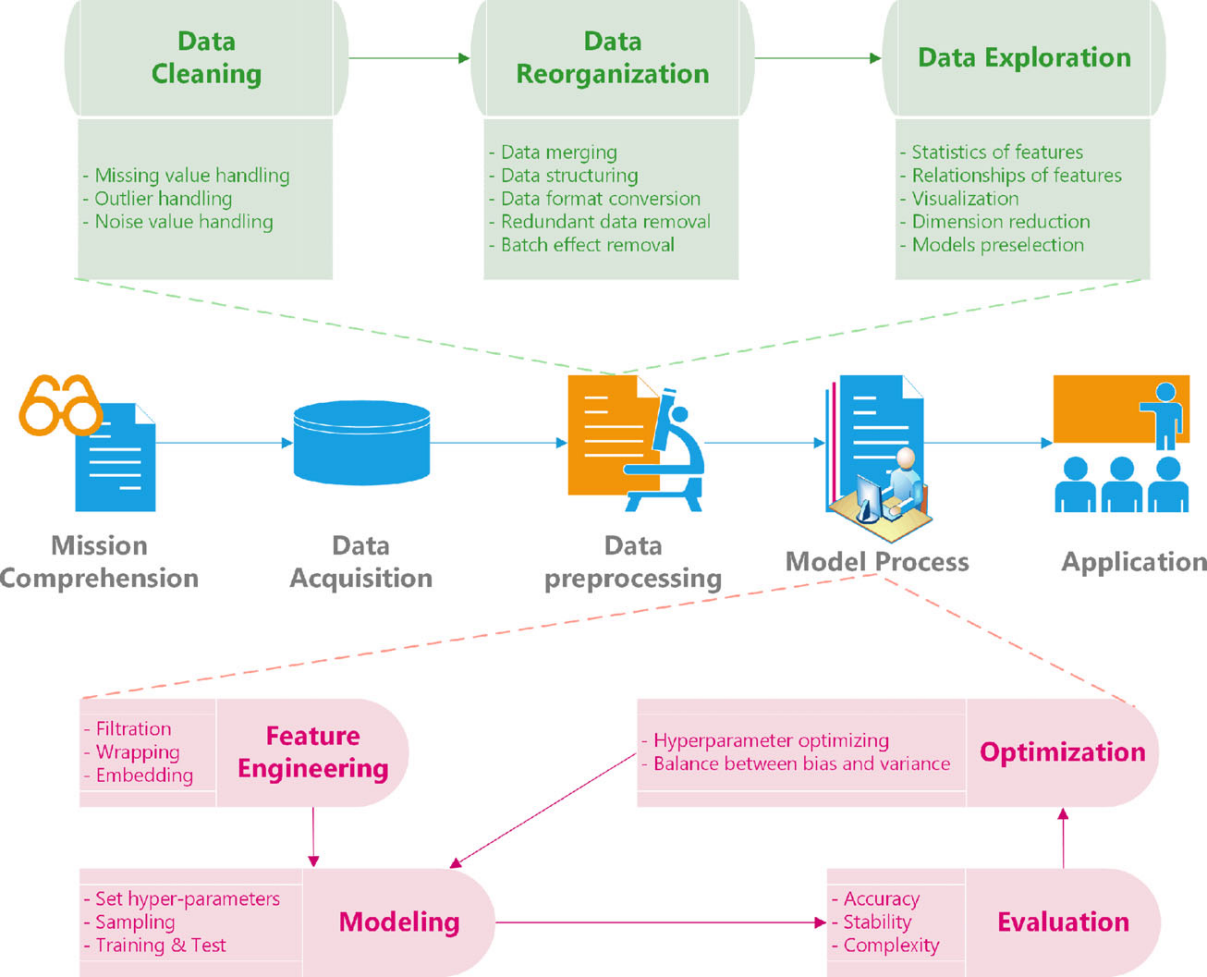
Data mining process of ctDNA detection. ctDNA, circulating tumor DNA.

Data mining has increasingly become a potential requirement for algorithm design of ctDNA detection. Given the rapid development in omics, the biological data in the open database online have increased exponentially, reforming the traditional data processing methods (39–41) (**Supplementary Figure 3**). By narrowing the scope of previous experimental data and using an appropriate workflow, the data mining system for ctDNA is simplified, accompanied by dramatically reduced costs of the whole research project. It provides the possibility for finding the new features of ctDNA hidden in the data structure to promote further development of ctDNA capture (**Figure 1**). For example, Misawa et al. mined the transcriptome data and filtered out the abnormal methylations as biomarkers in ctDNA, which assists in designing a mathematical model of ctDNA detection to identify patients with human papillomavirus–associated oropharyngeal cancer (42).

## Technical Obstacles and Potential Solutions of Data Processing in ctDNA Profiling

With the deepening understanding of biological features of ctDNA, the prevalence of ctDNA detection in cancer diagnosis inspired researchers. However, several technical shortcomings limit the clinical application of ctDNA detection.

Firstly, the sensitivity and specificity of ctDNA profiling are remarkably influenced by poor experimental conditions when facing complex biological characteristics (43). The trace amount and inevitable degradation of plasma ctDNA jeopardize ctDNA detection, especially when the blood sample is isolated and collected by centrifugation. Recent related studies demonstrate that plasma ctDNA loses about 50% after centrifugation (44, 45). The current blood storage manners are always accompanied by hemagglutination and extravasation, which considerably hamper ctDNA detection. Moreover, several commercial kits have been developed but show different extraction efficiencies and fragment size preferences, thereby challenging the repeatability and comparability of ctDNA detection resulting from different studies (46–49). Thus, the development of a universal standard protocol used for ctDNA extraction is essential in the future clinic application of ctDNA detection strategies.

Furthermore, the low signal-to-noise ratio remains a major problem for data processing of ctDNA detection. In addition to the low proportion of ctDNA in the serum cfDNA pools, as mentioned above, somatic mutations deriving from clonal hematopoietic (CH) and mitochondrial ctDNA also bring significant background noises. CH variants are cumulated with age, which could be attributed to the cloning expansion of stem cells carrying somatic mutations (50). Due to the high false-positive rate in ctDNA detection results, CH variants also interfere with the construction of the ctDNA mutation spectrum (51). Although mitochondrial ctDNA could be roughly excluded by size selection manner, the leaking information still exists during ctDNA detection (52). Given the above, the development of data analysis algorithms to increase signal-to-noise rate will facilitate the reliability of ctDNA as a tumor biomarker applied in clinical diagnosis. For instance, Nassiri et al. have developed a machine learning–based model to analyze the data generated from methylated HTS in ctDNA detection, increasing the accuracy of subtyping intracranial tumors (53). Moreover, CH variances produced by white blood cells are recognized by the combination of computational algorithms, then the false-positive rates of ctDNA detection would be decreased (54). With the help of statistical analysis and machine learning models, the CH variances spectrum can be built quickly and will be removed effectively by comparing it to the mutational spectrum of tumors constructed by ctDNA assays (30).

Additionally, the technological bias of HTS platforms inevitably interferes with the high-throughput ctDNA detection. These sequencing errors can be partially reduced or corrected. For instance, increased sequencing depth dilutes the error information (55), and the introduction of appropriate barcodes and indexes could evaluate the sequence duplication bias produced through polymerase chain reaction (PCR) amplification (56). The erroneous sequencing on barcodes, which affects the deduplication of unique ctDNA molecules and results in errors in aligning molecules to unique ctDNA, can be optimized by increasing the hamming edit distance between different barcodes (57). However, these additional barcodes occupy some part of reads and then reduce the actual length of target ctDNA to be sequenced, attributing to the limitation of the reading sequence length of long-read assembly technology. Therefore, the choice of proper barcodes used for HTS of ctDNA detection is the critical factor in resolving the inevitable sequencing errors, calling for new methods for pre- and post-sequencing error correction based on a statistical landscape.

## Clinical Relevance of Current ctDNA Profiling Technologies

The clinical relevance of the ctDNA biomarker shows substantial potential in non-invasive liquid biopsy, which may benefit millions of patients for early detection of tumor (58, 59), determination of tissue of origin (37), prediction of therapeutic effect, especially for immunotherapies (60–62), and monitoring (15, 30). The dynamic risk stratification correlated to the tumor genesis could also be facilitated by ctDNA detection such as occupation, age, living habits, and even mutational signatures (63). With the aid of classification or non-supervisor clustering, ctDNA detection technologies are conspicuously improved in terms of accuracy, sensitivity, specificity, operational convenience, and reasonable cost.

The patterns of mutational spectrums or epigenetic profiles recognized by data mining uncover the particular clinical relevance of ctDNA detection. The genome-wide mutational landscape is conducive to the evaluation of tumor mutation burden (13), neoplasm staging (64), genotyping (11), and the choice of therapies (65). Meanwhile, the methylation profiles of ctDNA contribute to discriminating patients from healthy cohorts (37), differentiating cancer types (53), and identifying the primary tumor location (66). These profiles can complement each other in many aspects, though those meaningful patterns below them require plenty of modeling theories for recognition accuracy. The combination of mutation and methylation spectrums makes the acquirement of detailed genomic landscapes possible, provides multiple insights into the tumor heterogeneity, and evaluates the impact of tumor heterogeneity on the selection of therapies, such as non-responders or drug resistance (67, 68).

In addition to its non-invasiveness, near real-time monitoring and prognosis prediction are additional advantages of ctDNA detection over tissue biopsy (69, 70). For example, the concentration of ctDNA was correlated with the prognosis of patients treated with pembrolizumab (10). Furthermore, ctDNA detection, as an auxiliary method for low-dose computed tomography, can track the molecular minimal residual disease and predict the risk of recurrence for tumor-free patients (18, 71). Finally, the real-time information of ctDNA detection reflects patients’ status and sheds light on the personalized profiling for each patient, which is essential for precision medicine (72).

## Future Directions Improving the Limit of Detection of ctDNA

There is a definite clue that an evolution is happening in high-throughput ctDNA detection by introducing novel sequencing platforms, a combination of different biomarkers, and a development of new principles. New-generation sequencing technologies, such as nanopore sequencing, begin to be utilized in ctDNA detections (73). Compared with HTS technologies, nanopore sequencing exhibits real-time sequencing and long reads, resulting in its potentially broad application in the field of nucleic acid sequencing in the future (74). Moreover, nanopore sequencing is PCR-free, avoiding amplification bias and errors of PCR during the process of sequencing library preparation. Although nanopore sequencing remains to have some shortcomings in sequencing short DNA fragments, many efforts have been made to ameliorate these shortcomings (2, 75). For instance, Sun et al. have applied the solid-state nanopore to detect ctDNA originating from serum samples. This strategy cooperates with the hybridization chain reaction to amplify the target’s signals, improve data authenticity, and overcome the hurdles of nanopore application (76).

Combining multi-biomarkers in liquid biopsy, based on optimized models and algorithms, has a higher efficiency in tumor detections. The various biomarkers used as the inputs of the detection model have a complementary function because of their different sensitivity and specificity to patients. For example, combining exosome RNA and ctDNA in plasma, Krug et al. leveraged the threshold of a predefined model to detect EGFR mutations in non-small cell lung cancer, achieving a higher sensitivity than that of ctDNA detection alone (77). Cohen et al. utilized the protein biomarkers as a supplement to ctDNA detection, and a few patients with ctDNA undetectable were finally detected (78). Furthermore, the combination of multi-biomarkers provides the convenience of multi-parameters to machine learning in future liquid biopsy and promotes the development of detection tools in the diagnosis and prognosis for patients with cancer.

New principles, whether biological feature-based or data-driven, are the catalyzers of the ctDNA detection improvement. In the last decades, discovering new principles of ctDNA detection methods contributes to promoting the clinical application of this intriguing biomarker of liquid biopsy (**Table 1**). For example, the definition of recurrence index, an index equal to total unique patients with mutations covered per kb of an exon, has been introduced into CAPP-Seq as a selection principle that obviously improved the limit of detection of ctDNA (34). The continuous evolution of new technical principles of data analysis provides the substantial potential to ctDNA as a promising biomarker for its future clinical utility (79).

**Table 1:** Comparison of techniques in ctDNA detection.

Techniques	Flux	Level	Sensitivity (%)	Specificity (%)	VAF or LOD (%)	Reference (PMID)
Lung-CLiP	HTS	somatic mutation	64, 82, and 100% for stages I, II, and III	98	0.01	32269342
iDES-enhanced CAPP-Seq	HTS	somatic mutation	90	96	0.02	27018799
CAPP-Seq	HTS	somatic mutation	50 for stage I, 100 for stages II–IV	96	0.02	24705333
TEC-Seq	HTS	somatic mutation	97.4	89	0.1	28814544
MCTA-Seq	HTS	methylation alteration	94	89	/	26516143
dPCR	PCR	somatic mutation	92.9	100	0.5	25324352
ARMS	PCR	somatic mutation	96.3	65.2	0.15	28868565
BEAMing	PCR	somatic mutation	90.4	93.5	0.001	28106345
MSP	PCR	methylation alteration	67	100	/	18006766

ctDNA, circulating tumor DNA; VAF, variant allele frequency; LOD, limit of detection. The symbol “/” means that we didn't found the exact data in that paper.

## Conclusion

Embracing data science, ctDNA is a promising biomarker in cancer detection. ctDNA has several exciting characteristics, which could be handled to raise strategies to improve ctDNA detection performance. Herein, we reviewed data science that played an essential role in current strategies, such as data selection, data mining, and data correction, to overcome the technical obstacles in ctDNA detection. The recognition of the value of directing data processing indicates a possible trend to exploit ctDNA assays further. With the rapid development of data acquisition methodologies, modeling, and data processing algorithms, ctDNA detection enhanced its prevalent advantages in monitoring intrinsic tumor information. Novel high-throughput technology platforms and the combination of diverse biomarkers in liquid biopsy were also essential for this technology advancement. ctDNA-based liquid biopsies, as an alternative or even a substitutive choice of tissue biopsy, have significant clinical relevance in cancer diagnosis and prognosis. Subsequent efforts should be continued to promote the advancement of the detection technologies, theories, and principles accelerated by prosperously developed data science.

## Author’s Note

The data of **Supplementary Figure 3** coming from the GEOdatabase, including the R script and Rdata file.

## Author Contributions

ML: writing—original draft, figure design, and visualization. SX: assistance with writing review and editing. CL: assistance with figure design and visualization. LiZ: necessary advice provision. LvZ: supervision, funding acquisition, writing review, and editing. All authors contributed to the article and approved the submitted version.

## Funding

This review was supported by grants from the National Natural Science Foundation of China (31500686, 31870855), the “Huxiang Young Talents Plan” Project of Hunan Province (2019RS2030), the Natural Science Foundation of Hunan Province (2020JJ5657), Fund for NUDT Young Innovator Awards (20190104), and Postgraduate Scientific Research Innovation Project of Hunan Province.

## Conflict of Interest

The authors declare that the research was conducted in the absence of any commercial or financial relationships that could be construed as a potential conflict of interest.

## Supplementary Material

Supplementary Figure 1 Origination of ctDNA. ctDNA, circulating tumor DNA; cfDNA, cell-free DNA.

Supplementary Figure 2 The workflow of CAPP-Seq. CAPP-Seq, the Cancer Personalized Profiling by deep sequencing; ctDNA, circulating tumor DNA; cfDNA, cell-free DNA.

Supplementary Figure 3 Rapid growth in the number of samples uploaded in the GEO (Gene Expression Omnibus) database.

## Abbreviations

CAPP-Seq, the Cancer Personalized Profiling by deep sequencing; CH, clonal hematopoietic; cfDNA, cell-free DNA; ctDNA, circulating tumor DNA; HTS, high-throughput sequencing; PCR, polymerase chain reaction.
