# Supplementary figures and images for: Application of Data Science in Circulating Tumor DNA Detection: A Promising Avenue Towards Liquid Biopsy
Source: Front Oncol. 2021 Jul 21;11:692322. doi: 10.3389/fonc.2021.692322 (PMC8337081; doi:10.3389/fonc.2021.692322)

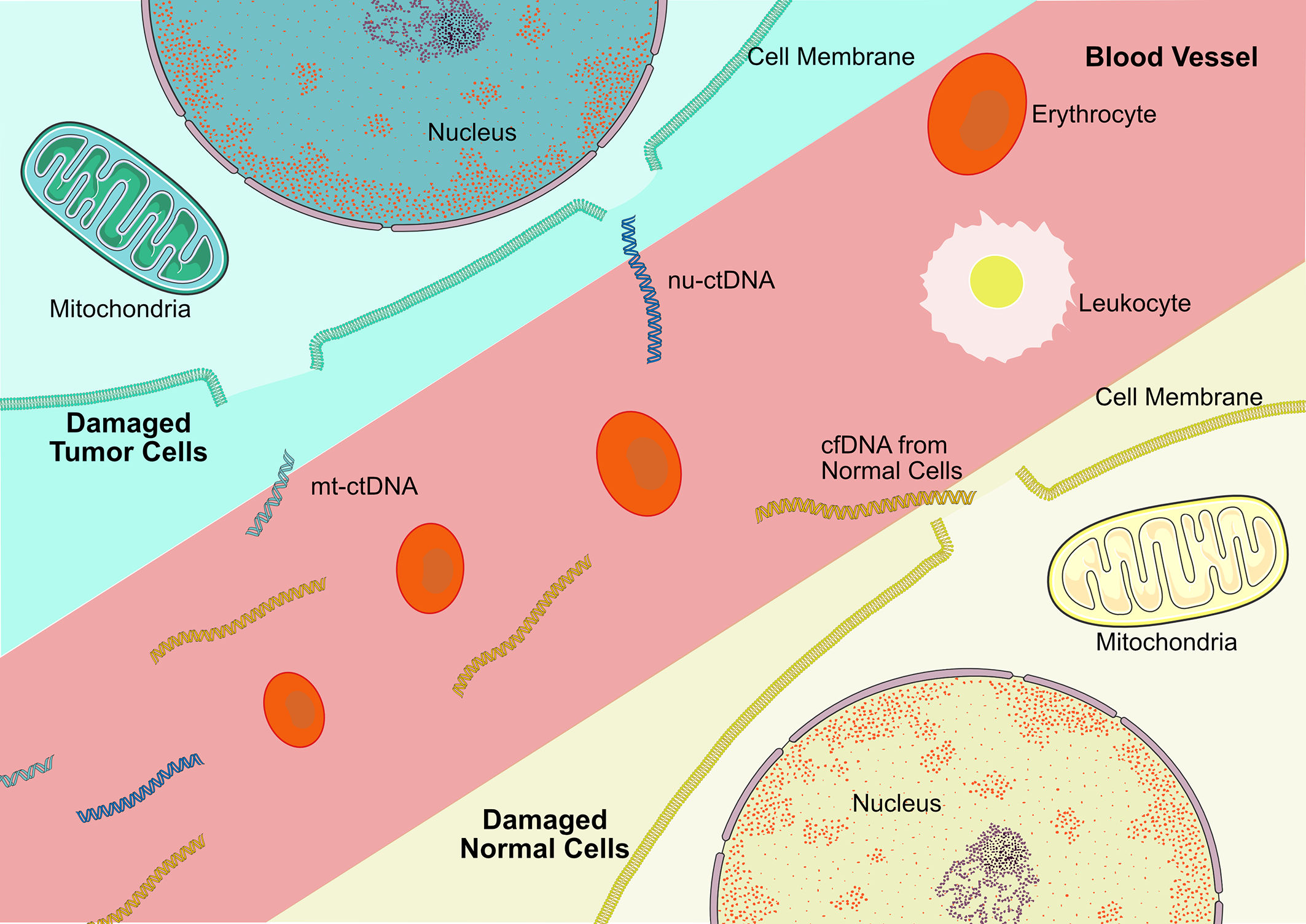

Supplement: Supplementary Figure 1 — Origination of ctDNA. ctDNA, circulating tumor DNA; cfDNA, cell-free DNA. [file Image_1.tiff]

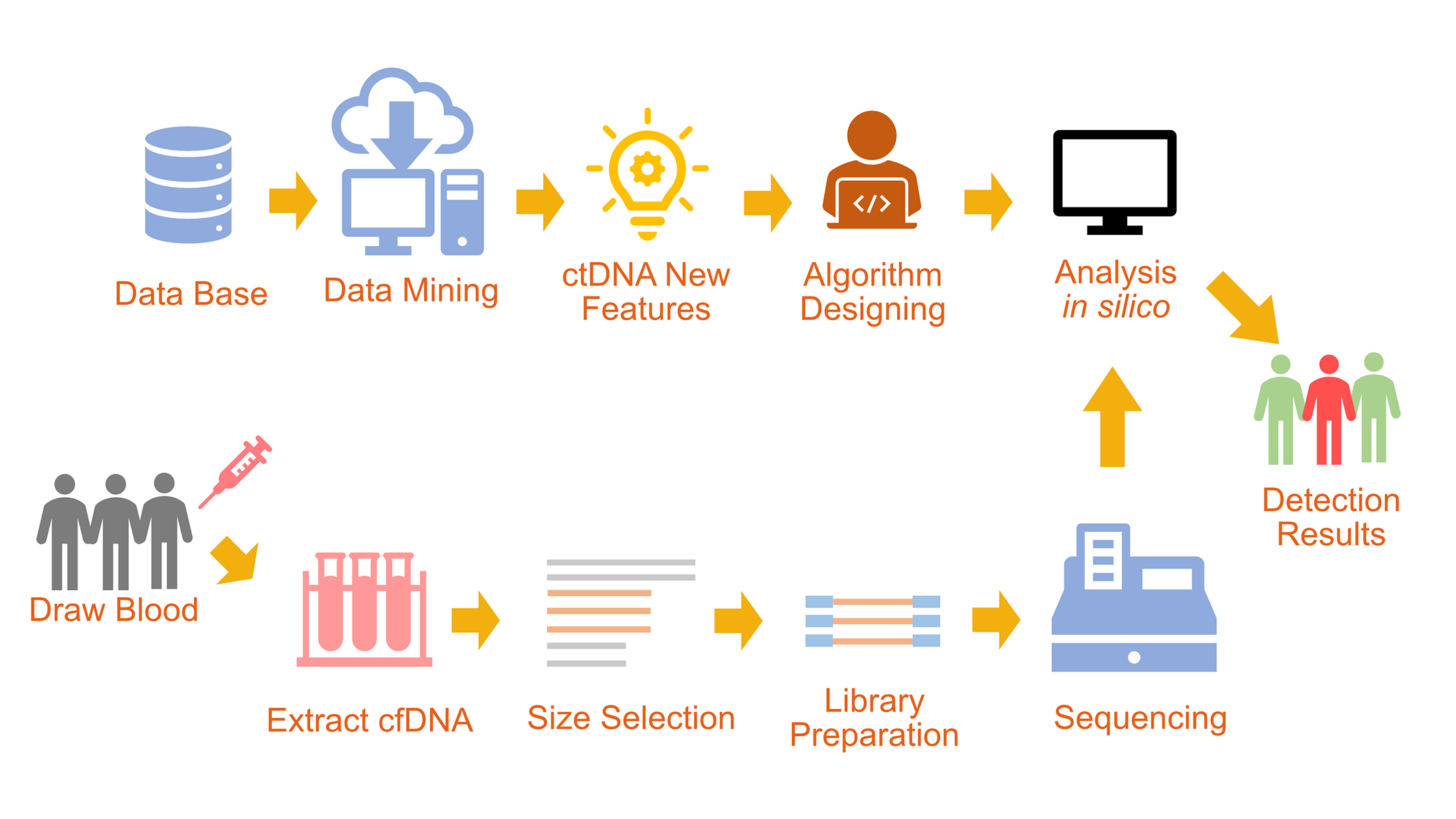

Supplement: Supplementary Figure 2 — The workflow of CAPP-Seq. CAPP-Seq, the Cancer Personalized Profiling by deep sequencing; ctDNA, circulating tumor DNA; cfDNA, cell-free DNA. [file Image_2.tiff]

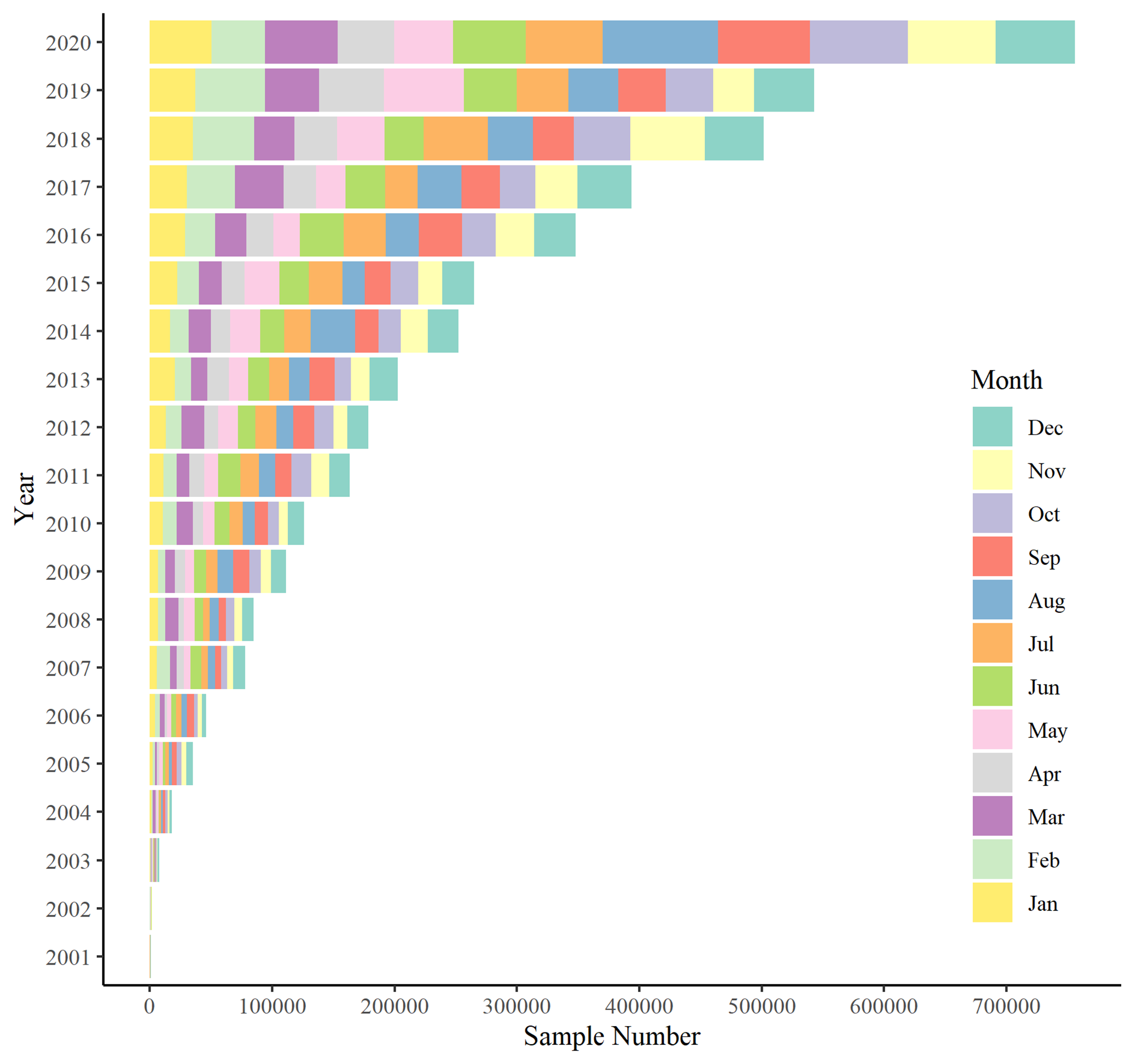

Supplement: Supplementary Figure 3 — Rapid growth in the number of samples uploaded in the GEO (Gene Expression Omnibus) database. [file Image_3.tiff]
